# Barriers to contraceptive implant removal among current and prior implant users in Kisumu, Kenya: Results from a cross-sectional population-based survey

**DOI:** 10.1371/journal.pgph.0005426

**Published:** 2025-11-13

**Authors:** Brooke W. Bullington, Stephanie Chung, Emilia Goland, Dickens Otieno Onyango, Leigh Senderowicz, Abigael Mwanyiro, Claire W. Rothschild, Ben Wekesa, Brian Frizzelle, Ginger Golub, Katherine Tumlinson

**Affiliations:** 1 Division of Family Planning, Department of Obstetrics & Gynecology, University of Utah, Salt Lake City, Utah, United States of America; 2 Department of Maternal and Child Health, Gillings School of Global Public Health, University of North Carolina at Chapel Hill, Chapel Hill, North Carolina, United States of America; 3 Product Development and Introduction, Global Health and Population, FHI 360, Durham, Chapel Hill, North Carolina, United States of America; 4 Kisumu County Department of Health, Kisumu, Kenya; 5 Department of Gender and Women’s Studies, University of Wisconsin, Madison, Wisconsin, United States of America; 6 Department of Obstetrics and Gynecology, School of Medicine and Public Health, University of Wisconsin, Madison, Wisconsin, United States of America; 7 Innovations for Poverty Action (IPA), Nairobi, Kenya; 8 Population Services International, Washington D.C., United States of America; 9 Carolina Population Center, University of North Carolina at Chapel Hill, Chapel Hill, North Carolina, United States of America; 10 Department of Health Policy and Management, Gillings School of Global Public Health, University of North Carolina at Chapel Hill, Chapel Hill, North Carolina, United States of America; African Population and Health Research Center, KENYA

## Abstract

There has been increased promotion of long-acting reversible contraception (LARC) in the Global South in recent years. Studies have documented that women face barriers to removal when they desire to discontinue their LARC method, though few have quantified how often such experiences occur. We estimated the proportion of current and prior implant users who experienced or anticipated challenges with method removal using secondary data from a population-based survey administered to reproductive-aged women (18–49 years) in Kisumu, Kenya. We included women who reported current contraceptive implant use (n = 518) or ever using an implant that was later removed (n = 838). We asked current implant users about their anticipated barriers to removal and experiences seeking removal. We asked those who reported prior implant removal about barriers they faced when seeking removal. Around 40% of current implant users anticipated barriers to implant removal. About 2% of current implant users had sought removal unsuccessfully in the last year, with cost being the most common barrier. Of those who had ever had an implant removed, 15% reported facing challenges with removal, with cost again being the most common barrier. Sociodemographic characteristics were not associated with facing challenges with removal. Many women anticipated or experienced barriers to implant removal. Cost was the most common barrier reported, highlighting the importance of eliminating fees associated with removal to ensure all women can discontinue their method when desired.

## Introduction

Long-acting reversible contraception (LARC), which includes contraceptive implants and intrauterine devices (IUDs), has been praised and prioritized by global family planning programs due to its safety, high efficacy with typical use, acceptability to users, and long durations of use [1,2]. The potential for low-cost and long-term contraceptive coverage has led to increased promotion of LARC-centric programs throughout the Global South, including the Implant Access Program and numerous programs to promote uptake of postpartum IUDs [3–5].

However, reproductive justice scholars have raised concerns that LARC methods present a threat to contraceptive autonomy, as they are “imposable,” defined by Clarke as being controlled by providers rather than the method user themself [1,6–8]. This is of special concern in the Global South, where LARC-first programs, which often promote LARC methods as the “best” contraceptive option, have resulted in increased uptake of LARC methods [9,10]. In fact, the implant has emerged as the most frequently used method in many countries, including Kenya [11,12]. Given presence of LARC-promoting programs across the Global South and the imposability of LARC methods, scholars have highlighted that researchers and family planning programs must identify and redress barriers to contraceptive autonomy in these settings [13,14].

Several prior studies have found that women face significant challenges when seeking LARC removal. In a qualitative analysis of women’s experiences with LARC removal in Kenya, women described their frustration with provider refusal to remove LARCs, pressure to keep their method for the full duration of its effectiveness, and the high cost of removal, even for expired methods [15]. Senderowicz also described refusal from providers to remove LARC methods as key to women’s experiences of contraceptive coercion, pointing to structural issues such as uptake targets and lack of provider training on removal as contributing factors [14]. However, few studies have explored how often women experience barriers to LARC removal. Using Performance Monitoring for Action (PMA) data from Burkina Faso and Kenya, Tumlinson and colleagues reported that the percentage of implant users experiencing unsuccessful removal between 2016 and 2020 declined from 9% to 2% in Kenya and from 7% to 3% in Burkina Faso – yet even with such reductions, thousands of women still remain unable to exercise reproductive autonomy in these countries due to removal barriers [11].

While available data and current literature provide estimates on the percent of current implant users experiencing barriers to removal, additional data is needed to understand how often both current and prior users face or have faced challenges to LARC removal, as well as the source and type of barrier encountered. Further, additional data are needed on the degree to which women anticipate barriers prior to attempting removal. It is possible that if women are concerned that they will face high barriers to removal services, anticipated barriers may prevent them from using their preferred method of contraception. In Kenya, prior research revealed that awareness of removal challenges resulted in some women avoiding contraceptive care at public facilities altogether [15].

Our objective with this analysis is to estimate the proportion of current and prior implant users who have experienced challenges with method removal, and to describe the specific challenges encountered when seeking method removal from a healthcare provider. We also seek to understand whether specific patient characteristics are correlated with barriers to method removal. Finally, we aim to understand the proportion of current users who anticipate experiencing barriers to method removal.

## Methods

### Ethics statement

Ethical review and approval for this study was provided by The Maseno University Ethics Review Committee (Reference number: MSU/DRP/MUERC/01054/22) in Kisumu, Kenya and the University of North Carolina at Chapel Hill (Study number: 21–2217).

### Parent study

This is a secondary analysis of data from a parent study implemented in Kisumu County, located in Western Kenya. The parent study was a cluster randomized controlled trial, which sought to evaluate the impact of two quality improvement interventions on contraceptive access and decision-making: a community score card and a citizen report card. Both interventions used a social accountability approach, and sought to assist communities in holding health systems accountable for their quality of contraceptive care. The parent study collected both individual and facility-level data related to contraceptive behavior and access both pre- and post-intervention. For the present analysis, we present only the pre-intervention (baseline) individual-level data, which were collected as a cross-sectional survey among women in Kisumu.

### Data collection

Data were collected among a representative sample of women, ages 18–49, residing in Kisumu. We first created mutually exclusive and exhaustive enumeration areas (EA) across the entire county. Next, within each EA we randomly selected building structures using publicly available data [16]. We visited selected structures and invited eligible women to participate in the study. We enrolled between 18–20 women in each EA, resulting in a final sample size of 2,484. For households containing more than one eligible woman, one was selected at random. Eligible women were approached by a trained female enumerator who explained the study and enrolled participants via a written informed consent protocol. The enumerator then collected data from each consenting woman using a structured questionnaire to ascertain demographic characteristics, reproductive histories, and contraceptive behaviors and access to care. Data were collected on a password protected and encrypted tablet using the Survey CTO platform.

### Measures

A total of 2,484 women ages 18–49 participated in the parent study’s women’s questionnaire. Among those, 2,149 were eligible for contraceptive use (i.e., not currently pregnant yet fecund). Among these 2,149, 1,301 reported current contraceptive use, with 518 reportedly using the contraceptive implant. Additionally, in the larger sample of 2,484 women, 838 report having ever used a contraceptive implant that was later removed. Our current analysis focuses on two overlapping subsamples of participants: first, women currently using implants (N = 518); and second, women who have ever had an implant removed in their lifetime (N = 838). Of note, 212 women are included in both subsamples (meaning they both currently use a contraceptive implant and previously used an implant that was removed).

Among the 518 women reporting current implant use, we implemented survey questions to estimate the proportion of women who were not told how to discontinue the implant at the time of method initiation, who anticipated a barrier to removing their implant, and who anticipated cost as a barrier to removal [17]. Among these same 518 women, we asked, “*At any time in the last 12 months, have you gone to the provider for any reason to ask them to remove your implant? Some reasons for this kind of visit might include experiencing bad side-effects, wanting to have a baby, or wanting to try a different method.”* We specified the 12 month time frame to improve recall bias. Among women with an affirmative response, we inquired where they went for removal services and why they were unsuccessful in achieving removal.

Among the 838 women reporting they ever used an implant that was later removed, we asked whether they experienced any challenges with implant removal. Among those who responded “yes,” we asked what challenges were encountered (select all that apply), the number of times they attempted removal, the type of facility (for both successful and unsuccessful removal attempts), whether they were charged a fee, and how much they were charged for implant removal.

### Statistical analysis

We estimated the proportion of women who reported experienced or anticipated barriers to removal, as well as the location and type of barrier experienced (and any associated fee), using simple descriptive statistics. We also investigated possible correlation between individual characteristics and barriers to implant removal using a chi squared tests for comparisons. All analyses were conducted in Stata version 17.

## Results

### Sociodemographic characteristics

A total of 518 women reported current implant use and 838 reported prior use of an implant that had since been removed. As illustrated by Table 1, most current implant users were married and had two or more children. Over half of implant users (54%) reported no formal education. About half (52%) lived in households with electricity, a proxy for socio-economic status. Similar patterns in sociodemographic characteristics were observed among the sample of women who had ever had an implant removed, though this group was older and had more children on average.

**Table 1:** Sociodemographic characteristics of current and prior implant users.

	Current implant users	Prior implant users
N	518	838
Age		
17-24	166 (32%)	165 (20%)
25-34	229 (44%)	418 (50%)
35-49	123 (24%)	255 (30%)
Marital status		
Married	402 (78%)	670 (80%)
Unmarried	116 (22%)	168 (20%)
Number of living children		
0-1	123 (24%)	115 (14%)
2-3	228 (44%)	378 (45%)
4-5	113 (22%)	248 (30%)
6+	54 (10%)	97 (12%)
Highest level of education completed		
None	281 (54%)	445 (53%)
Primary school	121 (23%)	228 (27%)
Secondary school or more	116 (22%)	165 (20%)
Household has electricity	269 (52%)	473 (56%)

### Removal knowledge and perceived barriers among current implant users

Among current contraceptive implant users (n = 518), many reported gaps in knowledge and perceived barriers to implant removal (Table 2). Two in five women (39%) said they were not informed about removal services at the time of insertion, and a similar proportion (39%) perceived that obtaining removal would be very difficult. Cost was also a concern, with 42% of implant users reporting it as a potential barrier to removal.

**Table 2:** Removal knowledge and perceived barriers to removal among current implant users.

	Current implant users	
	N = 518	
	n	%
Not informed about method removal at time of adoption	204	39%
Did not think they could get method removed without a lot of difficulty	201	39%
Thinks cost would be a barrier to removal	215	42%

### Unsuccessful removal attempts among current implant users

A small subset of current implant users (2%, n = 12) reported that they had unsuccessfully attempted to have their method removed in the past 12 months (Table 3). Eleven of the 12 (92%) sought removal in public facilities. Cost was the most frequently reported reason for unsuccessful removal (42%), followed by provider counselling against removal (17%). Other reasons included provider refusal, lack of qualified personnel, and being told to return later.

**Table 3:** Unsuccessful attempts to remove implant in 12 months among current implant users.

	Current implant users	
	N = 518	
	n	%
**Attempted to have implant removed**	12	2%
Among those who attempted removal (n = 12)		
**Reason implant was not removed**		
Facility not open	1	8%
Qualified provider not available	1	8%
Provider refused	1	8%
Cost of removal services	5	42%
Provider counseled against removal	2	17%
Told to return another day	1	8%
Referred elsewhere	1	8%
**Location where client sought removal**		
Public facility	11	92%
Private for profit	1	8%

### Barriers reported by past implant users

Among 2,061 women who reported ever using contraception, 838 (41%) reported ever having a contraceptive implant removed. Among this subgroup, 15% experienced at least one barrier when seeking removal services (Table 4). Similar to current users who unsuccessfully sought removal services, challenges were primarily facility- or provider-related. The most commonly reported barrier was cost of removal services (46%), followed by cases in which the provider attempted but was unable to remove the implant (26%). While the majority (92%) had their implant removed on the first attempt, 8% required two or more attempts. Most removal services – both unsuccessful (82%) and successful (76%) – occurred at public facilities. Notably, two-thirds of respondents reported paying a fee for the removal services, most commonly between 100 and 500 KES (Approximately 1–4 USD).

**Table 4:** Implant removal and barriers to removal among ever contraceptive users.

	Ever contraceptive users	
	N = 2,061	
	n	%
**Ever had an implant removed**	838	41%
Among those who have ever had an implant removed (n = 838)		
**Faced challenges having implant removed**	123	15%
**Challenges faced** (if faced challenges having implant removed, n = 123)		
Facility not open	2	2%
Qualified provider not available	11	9%
Provider attempted but could not remove	32	26%
Provider refused	3	2%
Cost of removal services	57	46%
Travel cost	7	6%
Provider counseled against removal	3	2%
Told to return another day	9	7%
Referred elsewhere	7	6%
It was buried deep within the arm	8	7%
Procedure caused complications	10	8%
Other	2	2%
**Number of removal attempts**		
1	773	92%
2	52	6%
3 or more	13	2%
**Location of unsuccessful removal** (if multiple removal attempts, n = 65)		
Public facility	53	82%
Private for profit	11	17%
Private not for profit	0	0%
Other	0	0%
Missing	1	2%
**Location of successful removal**		
Public facility	638	76%
Private for profit	193	23%
Private not for profit	3	0%
Other	4	0%
**Fee charged for removal**		
No fee	210	33%
10–50 KES	7	1%
51–100 KES	29	5%
101–200 KES	279	44%
201–500 KES	109	17%
More than 500 KES	4	1%

### Factors associated with barriers to removal

There were no clear patterns in sociodemographic characteristics comparing women who reported facing challenges having their implant removed and those who did not (Table 5). We found no associations between age, number of children, education or household electricity and facing challenges to implant removal. We did find a potential association between marital status and having faced challenges to implant removal, with a larger proportion of unmarried women in the group facing challenges compared to the group not facing challenges (26% vs. 19%, p = 0.07).

**Table 5:** Factors associated with barriers to implant removal among those who reported ever having an implant removed.

	Reported ever having an implant removed N = 838		
	Faced challenges having implant removed	Did not face challenges having implant removed	P value
N	123	715	
**Age**			0.7
17-24	22 (18%)	143 (20%)	
25-34	60 (49%)	358 (50%)	
35-49	41 (33%)	214 (30%)	
**Marital status**			0.07
Married	91 (74%)	579 (81%)	
Unmarried	32 (26%)	136 (19%)	
**Number of living children**			0.4
0-1	21 (17%)	94 (13%)	
2-3	54 (44%)	324 (45%)	
4-5	31 (25%)	217 (30%)	
6+	17 (14%)	80 (11%)	
**Highest level of education completed**			0.4
None	70 (57%)	375 (52%)	
Primary school	34 (28%)	194 (27%)	
Secondary school or more	19 (15%)	146 (20%)	
**Household has electricity**			0.9
Yes	69 (56%)	404 (57%)	
No	54 (44%)	311 (44%)	

## Discussion

This study reveals that a substantial proportion of reproductive-aged women in Kisumu, Kenya, both current or prior implant users, either anticipate or have already experienced barriers to method removal. Among current implant users, approximately 40% anticipated facing challenges when seeking removal, with many citing cost or provider resistance. Among participants who previously had an implant removed, 15% reported encountering at least one challenge during the process. Among those who reported ever having implant removal, individual sociodemographic characteristics such as age, marital status, and number of children were not associated with barriers to implant removal. Instead, barriers such as cost and provider behavior reflect structural limitations in service delivery and pose a threat to reproductive autonomy, signaling persistent gaps in rights-based contraceptive care.

Our findings align with existing studies that show that women around the world face numerous barriers when seeking to discontinue LARC. These barriers include provider refusal to remove methods, cost of removal services, lack of provider training in removal, shortages of necessary supplies for removal, or limited access to a facility equipped with the removal [11,14,15,18–20]. In the present study, cost was the most commonly reported barrier to removal among current and prior implant users. Of those who ever had their implant removed, two-thirds reported paying a fee, and more than 60% were charged at least 100 Kenyan shillings. Though 100 Kenyan shillings (approximately equivalent to 76 cents in USD) may appear to be a small fee, many people in Kenya live under the international poverty standard of $2.15 USD per day [21]; such costs can therefore be prohibitive. Our findings align with qualitative research from Kisumu, where women reported various costs associated with LARC removal. Although family planning services at public facilities in Kenya are officially free in Kenya, there remains ambiguity about whether this policy covers removal services [15]. We posit that many of the fees reported were informal, reflecting structural issues including facility-level funding constraints or lack of oversight that may require systemic solutions, including routine audits, stronger supervision mechanisms, and adequate and timely remuneration for health service employees [22]. Still, we echo prior calls for elimination of fees for LARC removal and increased transparency about contraceptive service charges in public facilities in Kenya [15].

This study builds on prior work from Sokol and colleagues, who quantified perceptions of access to LARC removal among women in Burkina Faso using data from 2017-18 [17]. The authors found that 13% of implant users were not informed about how to discontinue their method, 15% anticipated difficulty obtaining removal, and 17% expected cost to be a barrier to removal. In our study, which used the same survey items, we found that the proportion of implant users in Kisumu, Kenya anticipating barriers to removal was substantially higher, with approximately 40% reporting each of the aforementioned barriers. Prior research documenting widespread informal fees in Kenya’s public health sector may partially explain this difference [23]. Still, the high prevalence of anticipated barriers in both settings underscores the importance of measuring these indicators on population-based surveys to assess and address gaps in contraceptive autonomy. Additional efforts are needed to ensure LARC users can access removal services when desired.

Our results also show that individual characteristics, such as age, marital status, and number of children, were not associated with facing barriers to removal among those who previously had an implant removed. This aligns with prior research suggesting that outcomes related to contraceptive coercion, including provider-imposed contraceptive coercion at the time of method adoption and barriers to LARC removal, are structural rather than individual [14,24,25 ]. In other words, women’s experiences with removal barriers appear not to reflect provider biases against specific subgroups, but rather broader systematic norms that prioritize contraceptive continuation. These norms are rooted in the belief that contraceptive use is inherently beneficial, even when a user desires to stop contracepting. To eliminate barriers to LARC removal, it’s imperative to address the root cause of such beliefs and center reproductive autonomy in contraceptive programming and service delivery. This, accompanied by capacity-building to deal with deeply placed implants and complications related to implant removal, is an imperative step in eliminating barriers to LARC removal.

This study has several strengths, including the use of population-based data from reproductive-aged women in Kisumu, Kenya and the implementation of novel survey items to assess barriers to LARC removal. Several limitations should be noted. First, we measured participant sociodemographic characteristics at the time of survey and assessed whether those characteristics were associated with ever facing a barrier to removal among participants who reported prior implant removal. This may lead to potential misclassification of the exposure (i.e., the sociodemographic characteristics), particularly if a participant’s characteristics changed between the time their implant was removed and the time of survey. Second, due to the low prevalence of current and prior IUD use in our sample, we were unable to explore prior and anticipated barriers to IUD removal. Only a small number of current implant users (n = 12, 2%) reported having attempted to have their implant removed in the last year, making it difficult to draw broader conclusions about experiences with implant removal among this group. Additionally, this study focused on Kisumu county and findings may not be generalizable to other parts of Kenya. Finally, we used novel measures of anticipated barriers to implant removal that have not formally been validated. Future research should focus on refining and expanding measures of prior and anticipated barriers to LARC removal.

In conclusion, we found that 40% of current implant users in Kisumu, Kenya anticipated barriers to removal, and 15% of prior implant users faced a barrier to removal when they sought to discontinue their method. Cost was often cited as a barrier to removal, highlighting the urgent need to eliminate both formal and informal fees. Because barriers to LARC removal appear structural – driven by larger public health and demographic goals of eliminating unintended pregnancy rather than individual women’s characteristics – addressing them will require systems-level interventions. Shifting the focus of family planning programming away from promoting contraceptive use among all women and toward autonomous decision-making is an important step. Routine monitoring of removal fees and provider training that emphasizes agency in decision-making could also help reduce barriers to removal. Ultimately, ensuring that women can have their implants removed for free, without delay, and when they choose is essential for safeguarding reproductive autonomy.

## Supporting information

S1 Checklist Inclusivity in global research checklist. (DOCX)
